# Repeated craniotomies for intracranial tumors: is the risk increased? Pooled analysis of two prospective, institutional registries of complications and outcomes

**DOI:** 10.1007/s11060-018-03058-y

**Published:** 2018-11-24

**Authors:** Costanza Maria Zattra, David Y. Zhang, Morgan Broggi, Julia Velz, Flavio Vasella, Dominik Seggewiss, Silvia Schiavolin, Oliver Bozinov, Niklaus Krayenbühl, Johannes Sarnthein, Paolo Ferroli, Luca Regli, Martin N. Stienen

**Affiliations:** 1 Department of Neurosurgery, University Hospital Zurich and Clinical Neuroscience Center, University of Zurich, Frauenklinikstrasse 10, 8091 Zurich, Switzerland; 2 Neurosurgical Unit 2, Department of Neurosurgery, Fondazione IRCCS Istituto Neurologico Carlo Besta, Milan, Italy; 3 Public Health and Disability Unit, Department of Neurology, Fondazione IRCCS Istituto Neurologico Carlo Besta, Milan, Italy

**Keywords:** Brain tumor, Craniotomy, Complications, Mortality, Morbidity, Reoperation

## Abstract

**Purpose:**

Deciding whether to re-operate patients with intracranial tumor recurrence or remnant is challenging, as the data on safety of repeated procedures is limited. This study set out to evaluate the risks for morbidity, mortality, and complications after repeated operations, and to compare those to primary operations.

**Methods:**

Retrospective observational two-center study on consecutive patients undergoing microsurgical tumor resection. The data derived from independent, prospective institutional registries. The primary endpoint was morbidity at 3 months (M3), defined as significant decrease on the Karnofsky Performance Scale (KPS). Secondary endpoints were mortality, rate and severity of complications according to the Clavien–Dindo Grade (CDG).

**Results:**

463/2403 (19.3%) were repeated procedures. Morbidity at M3 occurred in n = 290 patients (12.1%). In univariable analysis, patients undergoing repeated surgery were 98% as likely as patients undergoing primary surgery to experience morbidity (OR 0.98, 95% CI 0.72–1.34, p = 0.889). In multivariable analysis adjusted for age, sex, tumor size, histology and posterior fossa location, the relationship remained stable (aOR 1.25, 95% CI 0.90–1.73, p = 0.186). Mortality was n = 10 (0.4%) at discharge and n = 95 (4.0%) at M3, without group differences. At least one complication occurred in n = 855, and the rate (35.5% vs. 35.9%, p = 0.892) and severity (CDG; p = 0.520) was similar after primary and repeated procedures. Results were reproduced in subgroup analyses for meningiomas, gliomas and cerebral metastases.

**Conclusions:**

Repeated surgery for intracranial tumors does not increase the risk of morbidity. Mortality, and both the rate and severity of complications are comparable to primary operations. This information is of value for patient counseling and the informed consent process.

**Electronic supplementary material:**

The online version of this article (10.1007/s11060-018-03058-y) contains supplementary material, which is available to authorized users.

## Introduction

Neurosurgeons strive to cure patients from intracranial tumors with a single operation, thus obviating the need for further interventions. Unfortunately, additional procedures are often necessary, mostly because of incomplete resection during the primary surgery, as well as recurrence. The question of whether to re-operate can be challenging, and many factors, including a patient’s clinical presentation and cancer prognosis, must be taken into consideration. In general, it is essential for therapeutic decision-making and informed consent to have robust estimates of both the benefits and the risks of a given treatment. This is particularly true in situations where therapeutic alternatives to surgery can be offered, such as radiotherapy and/or chemotherapy.

In daily clinical patient care, we experience both favorable and unfavorable effects of repeated surgeries for intracranial tumors. Intervening repeatedly is sometimes considered more difficult, as delicate anatomical structures have already been manipulated and both scarring and adhesions may interfere with the dissection [1]. Moreover, dural closure and wound healing may be more difficult, and infection rates higher. For these reasons, previous reports had pointed out higher morbidity and complication rates for repeated surgery [2–4]. On the other hand, the previously established surgical access may facilitate certain procedures, and thus support re-operation.

Given the scarcity of literature on this topic, we set out to investigate whether the risk profile of a repeated tumor resection is comparable to that of the initial procedure.

## Methods

### Study type and inclusion criteria

We performed a retrospective observational two-centre study, involving consecutive patients undergoing microsurgical resection of intracranial tumors via open craniotomy.

For this, we pooled institutional data from patients treated between 01/2013 and 12/2017 at the Department of Neurosurgery, University Hospital Zurich, Switzerland (USZ), and between 01/2014 and 12/2017 at the Department of Neurosurgery, Fondazione IRCCS Istituto Neurologico Carlo Besta Milan, Italy (IRCCS). Methodological details of the patient registries at USZ and IRCCS were described previously [5–7].

The sample was dichotomized into “repeated surgeries” (= study group; patients having undergone microsurgical resection of an intracranial tumor before) versus “primary surgeries” (control group; patients not having undergone microsurgical resection of an intracranial tumor before). Diagnostic biopsies were not considered as “surgery” in this context. Few patients of both groups might have undergone diagnostic sampling prior to surgery, to rule out lymphoma or non-neoplastic lesion (data not available). Also, patients operated via trans-sphenoidal access (TSS) were omitted from analysis, considering their distinct complication and outcome profile.

### Endpoints and recorded variables

The primary endpoint was postoperative morbidity, defined as a significant change on the Karnofsky Performance Scale (KPS) at 3-months follow-up (M3), compared to the preoperative status. As there is no established “minimum clinically important difference” of the KPS after neuro-oncological surgery and a 10-point change on the upper KPS is not as meaningful to a patient as a 10-point change on the lower KPS, we adapted the previous definition for “significant change” as a decrease of ≥ 20 points if baseline KPS ≥ 80, or a decrease of ≥ 10 points if baseline KPS < 80 [8]. The KPS scale was chosen for its close correlation to surgery-related outcomes and its predictive capacity for morbidity in intracranial tumor patients [6, 9].

Secondary endpoints were morbidity at discharge, mortality, length of hospitalization (LOH), as well as rate, type and severity of complications. Any deviation from the normal postoperative course was considered a complication, including adverse events that are sometimes expected (e.g. visual field deficit after resection of a glioma in the visual cortex), even when considered as acceptable or unavoidable in a clinical setting. Complications were classified according to two different systems: the Clavien–Dindo Grade [CDG; ranging from I (no intervention needed) to V (complication resulting in death; see Online Resource 1)] [10], and an etiological classification proposed by Ferroli et al. [7]: traumatic (i.e. directly related to the surgical manipulation), cerebrospinal fluid (CSF)-related (i.e. leaks, hydrocephalus), septic, hemorrhagic, ischemic, epileptic, general [non-central nervous system (CNS)] and other. In cases with multiple documented complications, the most severe one was chosen for statistical analysis.

Other variables recorded were: general patients’ demographics (age, sex), tumor type and size, eloquent and posterior fossa location. Eloquent areas were considered as motor, sensory, language or visual areas, hypothalamus, thalamus, internal capsule, brainstem, and pineal region [7]. Difficulty of tumor dissection was estimated by the need to manipulate major blood vessels and/or cranial nerves [7].

### Statistical analysis

Both demographic and disease-specific baseline information were described using frequencies and percentages for categorical variables. Ordinal variables were described as medians and interquartile range (IQR). Interval variables were described as group means and standard deviations (SD). Imbalances were tested using Pearson χ^2^ tests, Wilcoxon rank sum tests or student’s *t* tests, as appropriate.

Logistic regression analysis was performed to estimate the effect size of the relationship between the variable of interest “repeated surgery” and an endpoint. First, a univariable model was built to analyze the direct relationship. Then, a multivariable model was created to adjust for baseline group differences. Results were expressed as (adjusted) odds ratios ((a)OR) with 95% confidence intervals (CI), analyzed for changes after adjustment. Sensitivity analyses were performed. Since the question of safety of repeated operations is important to all tumor types, we combined data of patients suffering from a wide range of benign and malignant intracranial tumors for our main statistical model. We performed subgroup analyses, however, to explore the relationship between “repeated procedure” and morbidity separately for the three main histopathological tumor types. We decided against propensity score matching, as logistic regression was found to be superior for analyses with high numbers of events per confounder, such as ours [11].

In order to detect a statistically significant difference of 5% in the primary endpoint (M3 morbidity) with a power of 0.8 and alpha set at 0.05, a total of at least n = 1856 patients would be required with n = 429 in the study and n = 1427 in the control group. Statistical analyses were performed with Stata version 14.2 for Mac (College Station, TX: StataCorp LP). P-values < 0.05 were regarded as statistically significant.

### Ethical considerations

The scientific workup of registry data was approved by the institutional review boards of both institutions and the patient’s informed consent was waived. The authors report no relevant conflicts of interest.

## Results

A total of 2718 patients were identified, of which n = 315 patients undergoing TSS were omitted. Patient- and disease-specific information of the remaining 2403 patients [mean age 52.7 ± 17.7 years (SD); n = 1229 female (51.1%); 463 undergoing repeated surgery (19.3%)] is presented in Table 1. Patients undergoing repeated surgery were about 5 years younger, more often male and had slightly smaller tumors that were less often located in the posterior fossa. Repeated surgeries were most frequently performed for glioblastoma (26.2%), followed by meningioma (18.4%) and other histological subtypes (12.6%). Otherwise, study groups were balanced for baseline functional status and eloquent location, besides other surgical features (all p > 0.05; Table 1).

**Table 1:** Baseline table with patient demographics

	Primary surgery	Repeated surgery	p-value
Age in years; mean (SD)	53.8 (17.5)	48.4 (18.2)	< 0.001
Sex			0.004
Male	920 (47.4%)	254 (54.9%)	
Female	1020 (52.6%)	209 (45.1%)	
Histopathology			< 0.001
Meningioma	597 (29.9%)	85 (18.4%)	
Glioblastoma	423 (21.8%)	121 (26.2%)	
Adenoma	12 (0.6%)	8 (1.7%)	
Anapl. Astrocytoma	89 (4.6%)	48 (10.4%)	
Low grade glioma	117 (6.0%)	51 (11.0%)	
Metastasis	310 (16.0%)	47 (10.2%)	
Schwannoma	109 (5.6%)	15 (3.3%)	
(Epi-)dermoid	26 (1.3%)	7 (1.5%)	
Chordoma	8 (0.4%)	8 (1.7%)	
Craniopharyngioma	22 (1.1%)	14 (3.0%)	
Other	245 (12.6%)	58 (12.5%)	
Admission KPS; median (IQR)	90 (10)	90 (10)	0.756*
Tumor size in cm; mean (SD)	3.75 (1.8)	3.33 (1.6)	< 0.001^+^
Eloquent location			0.488
Yes	975 (50.3%)	241 (52.0%)	
No	965 (49.7%)	222 (48.0%)	
Posterior fossa location			0.009
Yes	415 (21.4%)	74 (16.0%)	
No	1525 (78.6%)	389 (84.0%)	
Major brain vessels manipulation			0.790
Yes	712 (36.7%)	173 (37.4%)	
No	1228 (63.3%)	290 (62.6%)	
Cranial nerve manipulation			0.931
Yes	499 (25.7%)	120 (25.9%)	
No	1441 (74.3%)	343 (74.1%)	
	n = 1940 (100%)	n = 463 (100%)	

Data is presented in mean [standard deviation (SD)], median [interquartile range (IQR)] or count (percent)

*Two-sample Wilcoxon rank-sum (Mann–Whitney) test used

^+^Two-sample *t* test used

### Analysis of the primary endpoint

Morbidity at M3 was registered in n = 290 patients (12.1%). In univariable analysis, patients undergoing repeated surgery were 98% as likely as patients undergoing primary surgery to experience morbidity. We are 95% confident that the likelihood ranges between 72 and 134% (OR 0.98, 95% CI 0.72–1.34, p = 0.889). In multivariable analysis, the adjusted relationship remained insignificant and with a small effect size (aOR 1.25, 95% CI 0.90–1.73, p = 0.186; Table 2). Sensitivity analyses indicated robustness of the model.

**Table 2:** Uni- and multivariate logistic regression analysis estimating the relationship between repeated microsurgical resection of an intracranial tumor and morbidity at M3

	Univariable analysis			Multivariable analysis		
	OR	95% CI	p-value	aOR	95% CI	p-value
Repeated surgery	0.98	0.72–1.34	0.889	1.25	0.90–1.73	0.186
Age (per year)	1.03	1.02–1.04	< 0.001	1.03	1.02–1.04	< 0.001
Male sex	1.54	1.20–1.97	0.001	1.47	1.13–1.90	0.004
Tumor size (per increase in category)	1.83	1.46–2.28	< 0.001	1.78	1.41–2.25	< 0.001
Histology	0.98	0.95–1.02	0.349	1.01	0.97–1.06	0.492
Posterior fossa location	1.05	0.78–1.42	0.757	1.39	1.00–2.25	0.047

The multivariable analysis is adjusted for baseline differences in age, sex, histological diagnosis, size and posterior fossa location of the tumor

Given the differences in the functional status of included patients with different histopathological tumor subtypes, subgroup analyses were made for those diagnosed with meningioma (n = 664, of which n = 85 repeated; Online Resource 2; aOR 0.70, 95% CI 0.20–2.41, p = 0.574), high- or low-grade glioma (n = 849, of which n = 220 repeated; Online Resource 3; aOR 1.42, 95% CI 0.91–2.23, p = 0.125) or cerebral metastasis (n = 357, of which n = 47 repeated; Online Resource 4; aOR 1.60, 95% CI 0.77–3.34, p = 0.207), respectively, with no significant difference in the morbidity risk observed.

### Analysis of the secondary endpoints

Morbidity at discharge was registered in n = 294 patients (12.2%). In both uni- (OR 0.94, 95% CI 0.68–1.28, p = 0.676) and multivariable analyses (aOR 1.06, 95% CI 0.77–1.47, p = 0.692; Table 3) the odds for morbidity at discharge were not increased in patients undergoing repeated surgery. The results were similar for the three histopathological tumor subtypes (see Online Resources 2–4).

**Table 3:** Logistic regression analysis estimating the relationship between repeated microsurgical resection of an intracranial tumor and morbidity at discharge

	Univariable analysis			Multivariable analysis		
	OR	95% CI	p-value	aOR	95% CI	p-value
Repeated surgery	0.94	0.68–1.28	0.676	1.07	0.77–1.47	0.692
Age (per year)	1.01	1.00–1.02	0.029	1.01	1.00–1.02	0.006
Male sex	1.21	0.95–1.55	0.124	1.19	0.93–1.53	0.171
Tumor size (per increase in category)	1.38	1.11–1.71	0.004	1.43	1.14–1.79	0.002
Histology	1.02	0.98–1.05	0.360	1.02	0.98–1.06	0.305
Posterior fossa location	1.58	1.20–2.09	0.001	1.76	1.31–2.35	< 0.001

The multivariable analysis is adjusted for baseline differences in age, sex, histological diagnosis, size and posterior fossa location of the tumor

Mortality was n = 10 (0.4%) at discharge, of which no death occurred after a repeated procedure (p = 0.122). Mortality was n = 95 (4.0%) at M3, of which 18 deaths occurred after a repeated procedure (p = 0.936). The odds for dying until 3 months were not increased in patients undergoing repeated surgery in both uni- (OR 0.98, 95% CI 0.58–1.65, p = 0.936) and multivariable analysis (aOR 1.33, 95% CI 0.77–2.29, p = 0.307; see Online Resource 5). In patients with intracranial meningiomas and cerebral metastases, those results could be reproduced (see Online Resources 2 and 4). M3 mortality was higher in patients with repeated glioma surgery, however (aOR 2.95, 95% CI 1.35–6.45, p = 0.007; see Online Resource 3).

In-hospital complications occurred in 855 (35.6%) patients, and both the rates (35.5% vs. 35.9%, p = 0.892) and severity according to the CDG were similar after primary and repeated procedures (p = 0.520; Table 4). The majority of complications in primary vs. repeated surgeries were CDG grade I (18.4% vs. 19.2%) and II (8.8% vs. 9.7%), therefore not requiring invasive treatment.

**Table 4:** Rate and severity of worst postoperative complication until discharge, according to the Clavien Dindo Grade (CDG).[10]

CDG	Primary surgery	Repeated surgery	p-value
None	1251 (64.5%)	297 (64.2%)	0.892
I	357 (18.4%)	89 (19.2%)	0.520
II	171 (8.8%)	45 (9.7%)	
III a	40 (2.1%)	11 (2.4%)	
III b	79 (4.1%)	18 (3.9%)	
IV a	27 (1.4%)	3 (0.6%)	
IV b	6 (0.3%)	− (0%)	
V	9 (0.4%)	− (0%)	
	n = 1940 (100%)	n = 463 (100%)	

The etiology of complications differed significantly between the two groups (Table 5; p = 0.005). In both groups, traumatic (15.7% vs. 14.0%), general medicine (6.2% vs. 4.5%) and CSF-related (2.8% vs. 4.3%) causes occurred most frequently. The relative frequency of CSF-related, ischemic, hemorrhagic complications, but especially epileptic complications (4.1% vs. 1.9%), was higher after repeated surgery (Table 5).

**Table 5:** Etiology of postoperative complication until discharge, according to the classification by Ferroli et al. [7]

Etiological category	Primary surgery	Repeated surgery	p-value
None	1251 (64.5%)	297 (64.2%)	0.892
Traumatic	304 (15.7%)	65 (14.0%)	0.005
CSF-related	55 (2.8%)	20 (4.3%)	
Septic	41 (2.1%)	2 (0.4%)	
Ischemic	55 (2.8%)	18 (3.9%)	
Hemorrhagic	53 (2.7%)	15 (3.2%)	
General medicine	120 (6.2%)	21 (4.5%)	
Epileptic	37 (1.9%)	19 (4.1%)	
Other	24 (1.2%)	6 (1.3%)	
	n = 1940 (100%)	n = 463 (100%)	

LOH was similar between patients undergoing primary (8.3 ± 7.7 days (SD)) and repeated surgery (7.9 ± 6.2 days (SD); p = 0.340).

## Discussion

Ongoing debates in neurosurgery focus on whether repeated procedures for the resection of intracranial tumors increase the risks for morbidity, mortality and postoperative complications. By analyzing a set of prospectively collected data from two independent neurosurgical departments with respect to this research question, we demonstrated that the risk for morbidity is not increased after repeated, as compared to primary microsurgical tumor resection (aOR 1.25, 95% CI 0.90–1.73, p = 0.186). We also found a low mortality rate in both groups, and similar rates and severity of postoperative complications. Those findings made in a large, heterogeneous cohort (n = 2403) could be reproduced in disease-specific subgroup analyses of patients with intracranial meningiomas (n = 664), high- or low-grade gliomas (n = 849) and cerebral metastases (n = 357).

Study groups were defined by dichotomizing the patient cohort into first-ever versus repeated tumor resection. Given the typical patient distribution of tertiary neuro-oncological referral centers, about one in five patients was operated for a second (or third) time. Since data of n = 2403 patients from two hospitals was pooled, a reasonably sized study group (n = 463; 19.3%) could be compared to n = 1940 controls. The found relationships are credible, owing to the typical characteristics of the study group (Table 1), internal consistency within the two independent datasets, and the results matching our personal clinical observations. The repeat-surgery sample consisted of the typical patient cohort that is prone to re-operations: younger patients with high-grade gliomas (HGG) or meningiomas of medium size that have kept a reasonably good functional status. Here again, the observed patient characteristics compare well with the expected ones, indicating validity of the analyzed data. The study and control groups were balanced for many baseline characteristics. Importantly, the admission KPS—a factor recently shown to influence postoperative mortality, morbidity and complications—was similar between the two groups [6]. The main statistical model was otherwise adjusted for variables that differed between the groups. Owing to the large sample size and robust endpoint, we can provide accurate estimates for the effect size of the relationship between “repeated surgery” and morbidity with tight confidence intervals for the main statistical model. The results were consistent in subgroup analyses for the most prevalent tumor subtypes and additional sensitivity analyses. Internal consistency is furthermore evident by similarity in the result of each of the analyzed outcome measures (morbidity, mortality, complication rate and severity, LOH).

When a surgeon has to choose whether or not to operate on a patient, he/she will balance the possible advantages with disadvantages in order to estimate the risks and benefits of the operation itself. This evaluation becomes more challenging in case of repeated surgical interventions, as their risks can be estimated less well.

In general surgery, there are advocates of an increased risk of repeated surgeries [12, 13], while others present more optimistic results [14, 15]. In neurosurgery, repeated procedures are most frequently performed for recurrence of intracranial tumors with a high tendency for re-growth despite macroscopically complete resection and/or adjuvant treatment. For HGG, atypical meningiomas or chordomas, for example, re-operation rates between 8–45% have been described [16–18]. Thus, besides reflecting upon the oncological benefit [17, 19–25], the surgeon must decide whether or not the safety of repeated procedures is given.

In theory, several aspects such as e.g. scarring of critical structures or prior dural opening can increase the difficulty of microsurgical tumor dissection or complicate dural closure. Moreover, wound closure defects/delay secondary to the use of adjuvant chemotherapy and/or radiotherapy [26, 27] and an increased rate of postoperative infections [3, 4] have been associated with repeated surgery. On the other hand, a previously established surgical corridor may also facilitate the access to the lesion. According to our own surgical experience, repeated operations are technically more difficult, but the present data indicate that careful patient care does not necessarily translate into worse outcomes, as discussed below.

In the literature, not much is present on the topic, with the majority of the articles focusing on HGGs owing to their high recurrence rates [22, 28].

Concerning the oncological benefit, the current literature indicates improved survival time in carefully selected patients undergoing repeated surgery [22, 23, 28–30]. Concerning the risks, Chang et al. found that there was a modest trend toward increased perioperative complications among patients undergoing repeated craniotomy, despite similar length of surgery [4]. In their cohort, there was also an increased risk for systemic infections and depression, while the rates of wound infections, seizures, thromboembolic or hemorrhagic complications were similar to those of patients undergoing primary surgeries. Since most patients remained in a neurologically stable or improved condition, the authors considered the safety of repeated surgeries acceptable [4]. Besides, the authors found preoperative KPS score and tumor size as predictive of outcome, which compares well to our previous and present results [6].

The evaluation of repeated surgery for other tumor types [e.g., low-grade gliomas (LGG), adenomas, metastases, meningiomas] is much less accounted for. However, an increase in overall survival and a moderate increase in risk emerge from the literature for recurrent adult [31–37] and pediatric brain tumors [26].

### Morbidity and mortality

Our present article adds important, prospectively collected data on complications and outcomes to the existing body of literature. Our rates of in-hospital mortality (0.0%; 0/463 procedures) and morbidity at discharge (11.7%; 54/463 procedures) were equally low after repeated as compared to primary surgery. Also after adjustment for baseline group differences, patients undergoing repeated surgery were as likely as patients undergoing primary surgery to experience discharge morbidity, showing no adverse effect of re-operations on the functional outcome immediately after the intervention. Independent factors that were found predictive of morbidity at discharge were posterior fossa location of the tumor (aOR 1.76, 95% CI 1.31–2.35), increase in tumor size category (aOR 1.43, 95% CI 1.14–1.79) and higher age (aOR 1.01, 95% CI 1.00–1.02; Table 3). Those findings are not surprising, as the posterior fossa contains vital structures such as the brainstem, but also dissecting tumors in the vicinity of cranial nerves is more likely to result in morbidity. Posterior fossa surgery is known to have an increased rate of complications compared to supra-tentorial surgery, in particular CSF-leakage [38]. Moreover, the removal of larger tumors usually results in more affection of the surrounding vital brain tissue and those challenging procedures are known to have more impact on elder patients [39]. The fact that those well-known observations can be detected in our analysis, however, lends credibility to the underlying dataset. Disease-specific subgroup analyses confirmed the results (see Online Resources 2–4), with stable effect sizes indicating a similar risk of first-ever or repeated craniotomies for intracranial meningiomas, high- and low-grade gliomas and cerebral metastases at time of discharge.

The results at discharge are particularly interesting in this regard, as they relate more directly to the surgical procedure, whereas the M3 follow-up results may already be influenced by the natural disease course. Accordingly, despite a similar likelihood for morbidity and mortality after repeated procedures for the complete cohort (see Table 2 and Online Resource 5), we noticed higher odds for M3 mortality in glioma patients who underwent repeated surgery. Since there was no discharge mortality in patients undergoing repeated surgery and discharge morbidity was similar in glioma patients (see Online Resource 3), the higher M3 mortality in this patient cohort is likely more related to disease progression than to the surgery itself. Repeated surgical procedures in patients with high-grade gliomas sometimes represent “salvage procedures”, when options with radiation therapy and chemotherapy have been exhausted. The effect of this group with particularly dismal prognosis can be appreciated in our dataset.

### Complications and LOH

Previous studies have demonstrated an increased risk for perioperative complications after repeated neurosurgery [4, 26, 40]. However, these works included smaller patient cohorts, and none used validated grading systems for complications. In our series, both the overall rate of complications, as well as their severity according to the CDG was comparable. The majority of the complications both in the primary and in the repeated surgery groups were minor (grade I or II). Besides the methodological differences of our study compared to the previous ones [4, 26, 40], our more favorable results in repeated procedures could be due to increased awareness for the challenges of repeated surgery, as well as recent advances in neurosurgical techniques, neuro-anesthesia and neuro-critical care [4, 41].

Our analysis showed some differences in the etiology of complications between the study groups, with CSF-related, ischemic/hemorrhagic and epileptic complications being increased in the repeated surgery group. In particular the relative frequency of seizures after repeated operations was twice as high as the one following primary surgery (4.1% vs. 1.9%). This finding could be indicative of more irritable or vulnerable brain parenchyma after repeated surgery. The marginally higher rate of CSF-related complications could have a number of explanations, including previous opening and re-adaptation of the dura and the need for autologous or allogeneic dural substitute for closure. More research is needed, however, to confirm these observations. As only the complication with highest CDG was recorded, the present data do not allow for the calculation of complication-specific incidence rates.

### Strengths and limitations

Strengths include the prospective collection of large and independent datasets from two hospitals in different countries, which reduces bias, as management strategies varied, and allows for generalization to other centers. Variable definitions and the design of both patient registries were unified. We chose defined outcomes and endpoints, and intentionally applied simple but adequate statistical approaches to address a pre-specified hypothesis with the least amount of statistical tests in order to avoid type-I errors. Given the relatively large sample size, the study had enough power to exclude a between-group difference of 5% in the primary endpoint.

Limitations include cohort heterogeneity, especially concerning the histopathological diagnosis, which is why disease-specific subgroup analyses are provided. Also, selection bias may apply and the results should not be generalized to the whole populations of patients presenting with recurrent tumors. Once a patient presents with tumor recurrence, surgeons tend to propose surgery as primary treatment to patients that have a reasonable chance to obtain a favorable outcome (higher KPS, younger age, etc.). On the contrary, for those patients deemed unsuitable for surgery alternative treatment (radiosurgery, radiotherapy, chemotherapy) is more frequently considered. Patients undergoing repeated surgery might therefore be subject to a survival bias. As for answering this particular research question a randomized controlled trial is not possible, best evidence will need to derive from retrospective analysis of prospective, high-quality databases. Results must be interpreted within the limitations of its design. Moreover, as we did not analyze long-term survival, we could not estimate the oncological benefit of repeated operations. Additional analyses of the impact of adjuvant radio- and chemotherapy on the risk for postoperative complications would have been interesting; future studies should explore these issues.

## Conclusions

Repeated surgical procedures for intracranial tumors in general do not increase the risk of morbidity in carefully selected candidates, compared to primary surgeries. The rate and severity of complications is comparable, and mortality is equally low. Those findings made in a large, heterogeneous cohort could be reproduced in disease-specific subgroup analyses of patients with intracranial meningiomas, gliomas and cerebral metastases. When considering repeated surgery, this information will be of value for patient counseling and the informed consent process.

## Electronic supplementary material

Below is the link to the electronic supplementary material.

Supplementary material 1 (DOC 82 KB)

Online Resource 1 (PDF 62 KB)

Online Resource 2 (PDF 80 KB)

Online Resource 3 (PDF 85 KB)

Online Resource 4 (PDF 85 KB)

Online Resource 5 (PDF 73 KB)
