# Supplementary material for: Repeated craniotomies for intracranial tumors: is the risk increased? Pooled analysis of two prospective, institutional registries of complications and outcomes
Source: J Neurooncol. 2018 Nov 24;142(1):49–57. doi: 10.1007/s11060-018-03058-y (PMC6399174; doi:10.1007/s11060-018-03058-y)
Supplement: Supplementary file 2 — Online Resource 1 (PDF 62 KB) [file 11060_2018_3058_MOESM2_ESM.pdf]

Online Resource 1: According to the Clavien-Dindo Grade (CDG),[10] complications are classified into five grades based on the treatment required to manage them:

I) Any deviation from the normal course, but not requiring any intervention (other than anti-emetics, antipyretics, analgesics, diuretics, electrolytes and physical therapy

II) Any deviation requiring pharmacological treatment other than that of grade I and/or transfusions and/or total parenteral nutrition

III) Complications requiring surgical, endoscopic or radiological intervention (3a= not under general anesthesia; 3b=under general anesthesia)

IV) Life-threatening complications requiring Intensive Care Unit management (4a=single organ dysfunction; 4b=multi-organ dysfunction)

V) Complications causing death of the patient within 30 days of surgery.

This online supplementary document is part of the article “Repeated craniotomies for intracranial tumors: is the risk increased? Pooled analysis of two prospective, institutional registries of complications and outcomes” by

Costanza Maria Zattra[1,2], MD; David Y. Zhang[1], MSc; Morgan Broggi[2], MD, PhD; Julia Velz[1], MD; Flavio Vasella[1], MD; Dominik Seggewiss[1], MD; Silvia Schiavolin[3], PsyD; Oliver Bozinov[1], MD; Niklaus Krayenbühl[1], MD; Johannes Sarnthein[1], PhD; Paolo Ferroli[2], MD; Luca Regli[1], MD; Martin N. Stienen[1], MD, FEBNS

[1]Department of Neurosurgery, University Hospital Zurich and Clinical Neuroscience Center, University of Zurich, Frauenklinikstrasse 10, 8091 Zurich, Switzerland

[2]Neurosurgical Unit 2, Department of Neurosurgery, Fondazione IRCCS Istituto Neurologico Carlo Besta, Milan, Italy

[3]Public Health and Disability Unit, Department of Neurology, Fondazione IRCCS Istituto Neurologico Carlo Besta, Milan, Italy

Correspondence to:
Martin N. Stienen, MD / FEBNS
Department of Neurosurgery
University Hospital Zurich & Clinical Neuroscience Center
University of Zurich
Frauenklinikstrasse 10
8091 Zurich, Switzerland
Tel: +41 – (0)44 – 255 – 1111
Email: mnstienen@gmail.com
