# Supplementary material for: Repeated craniotomies for intracranial tumors: is the risk increased? Pooled analysis of two prospective, institutional registries of complications and outcomes
Source: J Neurooncol. 2018 Nov 24;142(1):49–57. doi: 10.1007/s11060-018-03058-y (PMC6399174; doi:10.1007/s11060-018-03058-y)
Supplement: Supplementary file 3 — Online Resource 2 (PDF 80 KB) [file 11060_2018_3058_MOESM3_ESM.pdf]

Online Resource 2: Subgroup analysis on patients undergoing microsurgical resection of an intracranial meningioma. The data is based on 664 patients, of which 85 underwent repeated surgery.

Logistic regression analysis estimating the relationship between repeated microsurgical resection of an intracranial meningioma and morbidity three months after surgery. The multivariate analysis is adjusted for baseline differences in age, sex, size and posterior fossa location of the tumor.

**M3 morbidity**	**Univariate analysis**			**Multivariate analysis**		
	**OR**	**95% CI**	**p-value**	**OR**	**95% CI**	**p-value**
Repeated surgery	0.63	0.19 – 2.09	0.446	0.70	0.20 – 2.41	0.574
Age (per year)	-	-	-	1.01	0.99 – 1.05	0.294
Male sex	-	-	-	1.92	0.93 – 3.98	0.078
Tumor size (per increase in category)	-	-	-	1.74	0.92 – 3.31	0.090
Posterior fossa location	-	-	-	3.30	1.58 – 6.89	0.001

Logistic regression analysis estimating the relationship between repeated microsurgical resection of an intracranial meningioma and morbidity at time of hospital discharge. The multivariate analysis is adjusted for baseline differences in age, sex, size and posterior fossa location of the tumor.

**Discharge morbidity**	**Univariate analysis**			**Multivariate analysis**		
	**OR**	**95% CI**	**p-value**	**OR**	**95% CI**	**p-value**
Repeated surgery	1.05	0.48 – 2.30	0.897	1.20	0.54 – 2.68	0.657
Age (per year)	-	-	-	1.01	0.99 – 1.03	0.593
Male sex	-	-	-	1.52	0.85 – 2.71	0.159
Tumor size (per increase in category)	-	-	-	1.75	1.06 – 2.89	0.027
Posterior fossa location	-	-	-	2.81	1.56 – 5.07	0.001

As there was no 3-month mortality in patients undergoing repeated microsurgical resection of an intracranial meningioma, logistic regression analysis estimating the relationship between repeated surgery and mortality could not be calculated.

This online supplementary document is part of the article “Repeated craniotomies for intracranial tumors: is the risk increased? Pooled analysis of two prospective, institutional registries of complications and outcomes” by Costanza Maria Zattra[1,2], MD; David Y. Zhang[1], MSc; Morgan Broggi[2], MD, PhD; Julia Velz[1], MD; Flavio Vasella[1], MD; Dominik Seggewiss[1], MD; Silvia Schiavolin[3], PsyD; Oliver Bozinov[1], MD; Niklaus Krayenbühl[1], MD; Johannes Sarnthein[1], PhD; Paolo Ferroli[2], MD; Luca Regli[1], MD; Martin N. Stienen[1], MD, FEBNS

[1]Department of Neurosurgery, University Hospital Zurich and Clinical Neuroscience Center, University of Zurich, Frauenklinikstrasse 10, 8091 Zurich, Switzerland
[2]Neurosurgical Unit 2, Department of Neurosurgery, Fondazione IRCCS Istituto Neurologico Carlo Besta, Milan, Italy
[3]Public Health and Disability Unit, Department of Neurology, Fondazione IRCCS Istituto Neurologico Carlo Besta, Milan, Italy

Correspondence to:
Martin N. Stienen, MD / FEBNS
Department of Neurosurgery
University Hospital Zurich & Clinical Neuroscience Center
University of Zurich
Frauenklinikstrasse 10
8091 Zurich, Switzerland
Tel: +41 – (0)44 – 255 – 1111
Email: mnstienen@gmail.com
