# Supplementary material for: Repeated craniotomies for intracranial tumors: is the risk increased? Pooled analysis of two prospective, institutional registries of complications and outcomes
Source: J Neurooncol. 2018 Nov 24;142(1):49–57. doi: 10.1007/s11060-018-03058-y (PMC6399174; doi:10.1007/s11060-018-03058-y)
Supplement: Supplementary file 4 — Online Resource 3 (PDF 85 KB) [file 11060_2018_3058_MOESM4_ESM.pdf]

Online Resource 3: Subgroup analysis on patients undergoing microsurgical resection of a high- or low-grade glioma. The data is based on 849 patients, of which 220 underwent repeated surgery.

Logistic regression analysis estimating the relationship between repeated microsurgical resection of a high- or low-grade glioma and morbidity three months after surgery. The multivariate analysis is adjusted for baseline differences in age, sex, size and posterior fossa location of the tumor.

**M3 morbidity**	**Univariate analysis**			**Multivariate analysis**		
	**OR**	**95% CI**	**p-value**	**OR**	**95% CI**	**p-value**
Repeated surgery	0.89	0.59 – 1.35	0.592	1.42	0.91 – 2.23	0.125
Age (per year)	-	-	-	1.04	1.02 – 1.05	<0.001
Male sex	-	-	-	0.93	0.63 – 1.37	0.708
Tumor size (per increase in category)	-	-	-	2.00	1.41 – 2.85	<0.001
Posterior fossa location	-	-	-	2.44	1.07 – 5.60	0.035

Logistic regression analysis estimating the relationship between repeated microsurgical resection of a high- or low-grade glioma and morbidity at time of hospital discharge. The multivariate analysis is adjusted for baseline differences in age, sex, size and posterior fossa location of the tumor.

**Discharge morbidity**	**Univariate analysis**			**Multivariate analysis**		
	**OR**	**95% CI**	**p-value**	**OR**	**95% CI**	**p-value**
Repeated surgery	0.73	0.46 – 1.15	0.175	0.89	0.55 – 1.44	0.625
Age (per year)	-	-	-	1.01	1.00 – 1.03	0.027
Male sex	-	-	-	1.16	0.77 – 1.75	0.481
Tumor size (per increase in category)	-	-	-	1.55	1.08 – 2.21	0.017
Posterior fossa location	-	-	-	1.23	0.51 – 2.93	0.645

Logistic regression analysis estimating the relationship between repeated microsurgical resection of a high- or low-grade glioma and mortality three months after surgery. The multivariate analysis is adjusted for baseline differences in age, sex, size and posterior fossa location of the tumor.

M3 mortality	Univariate analysis			Multivariate analysis		
	**OR**	**95% CI**	**p-value**	**OR**	**95% CI**	**p-value**
Repeated surgery	1.45	0.71 – 2.96	0.302	2.95	1.35 – 6.45	0.007
Age (per year)	-	-	-	1.06	1.03 – 1.08	<0.001
Male sex	-	-	-	0.69	0.34 – 1.40	0.708
Tumor size (per increase in category)	-	-	-	2.65	1.31 – 5.33	0.007
Posterior fossa location	-	-	-	1.13	0.14 – 9.11	0.908

This online supplementary document is part of the article "Repeated craniotomies for intracranial tumors: is the risk increased? Pooled analysis of two prospective, institutional registries of complications and outcomes" by Costanza Maria Zattra[1,2], MD; David Y. Zhang[1], MSc; Morgan Broggi[2], MD, PhD; Julia Velz[1], MD; Flavio Vasella[1], MD; Dominik Seggewiss[1], MD; Silvia Schiavolin[3], PsyD; Oliver Bozinov[1], MD; Niklaus Krayenbühl[1], MD; Johannes Sarnthein[1], PhD; Paolo Ferroli[2], MD; Luca Regli[1], MD; Martin N. Stienen[1], MD, FEBNS

[1]Department of Neurosurgery, University Hospital Zurich and Clinical Neuroscience Center, University of Zurich, Frauenklinikstrasse 10, 8091 Zurich, Switzerland

[2]Neurosurgical Unit 2, Department of Neurosurgery, Fondazione IRCCS Istituto Neurologico Carlo Besta, Milan, Italy

[3]Public Health and Disability Unit, Department of Neurology, Fondazione IRCCS Istituto Neurologico Carlo Besta, Milan, Italy

Correspondence to:

Martin N. Stienen, MD / FEBNS
Department of Neurosurgery
University Hospital Zurich & Clinical Neuroscience Center
University of Zurich
Frauenklinikstrasse 10
8091 Zurich, Switzerland
Tel: +41 – (0)44 – 255 – 1111
Email: mnstienen@gmail.com
