# Supplementary material for: Repeated craniotomies for intracranial tumors: is the risk increased? Pooled analysis of two prospective, institutional registries of complications and outcomes
Source: J Neurooncol. 2018 Nov 24;142(1):49–57. doi: 10.1007/s11060-018-03058-y (PMC6399174; doi:10.1007/s11060-018-03058-y)
Supplement: Supplementary file 5 — Online Resource 4 (PDF 85 KB) [file 11060_2018_3058_MOESM5_ESM.pdf]

Online Resource 4: Subgroup analysis on patients undergoing microsurgical resection of a cerebral metastasis. The data is based on 357 patients, of which 47 underwent repeated surgery.

Logistic regression analysis estimating the relationship between repeated microsurgical resection of a cerebral metastasis and morbidity three months after surgery. The multivariate analysis is adjusted for baseline differences in age, sex, size and posterior fossa location of the tumor.

**M3 morbidity**	**Univariate analysis**			**Multivariate analysis**		
	**OR**	**95% CI**	**p-value**	**OR**	**95% CI**	**p-value**
Repeated surgery	1.40	0.69 – 2.86	0.355	1.60	0.77 – 3.34	0.207
Age (per year)	-	-	-	1.01	0.98 – 1.03	0.540
Male sex	-	-	-	1.84	1.08 – 3.14	0.026
Tumor size (per increase in category)	-	-	-	1.21	0.72 – 2.01	0.475
Posterior fossa location	-	-	-	1.38	0.74 – 2.55	0.311

Logistic regression analysis estimating the relationship between repeated microsurgical resection of a cerebral metastasis and morbidity at time of hospital discharge. The multivariate analysis is adjusted for baseline differences in age, sex, size and posterior fossa location of the tumor.

**Discharge morbidity**	**Univariate analysis**			**Multivariate analysis**		
	**OR**	**95% CI**	**p-value**	**OR**	**95% CI**	**p-value**
Repeated surgery	1.74	0.67 – 4.52	0.253	1.53	0.57 – 4.09	0.401
Age (per year)	-	-	-	1.02	0.99 – 1.06	0.243
Male sex	-	-	-	1.10	0.51 – 2.39	0.809
Tumor size (per increase in category)	-	-	-	0.62	0.29 – 1.30	0.204
Posterior fossa location	-	-	-	0.56	0.18 – 1.69	0.301

Logistic regression analysis estimating the relationship between repeated microsurgical resection of a cerebral metastasis and mortality three months after surgery. The multivariate analysis is adjusted for baseline differences in age, sex, size and posterior fossa location of the tumor.

M3 mortality	Univariate analysis			Multivariate analysis		
	**OR**	**95% CI**	**p-value**	**OR**	**95% CI**	**p-value**
Repeated surgery	0.59	0.20 – 1.74	0.342	0.66	0.22 – 1.98	0.462
Age (per year)	-	-	-	1.02	0.99 – 1.05	0.133
Male sex	-	-	-	1.58	0.83 – 3.00	0.164
Tumor size (per increase in category)	-	-	-	0.93	0.50 – 1.75	0.833
Posterior fossa location	-	-	-	1.75	0.86 – 3.56	0.125

This online supplementary document is part of the article "Repeated craniotomies for intracranial tumors: is the risk increased? Pooled analysis of two prospective, institutional registries of complications and outcomes" by Costanza Maria Zattra[1,2], MD; David Y. Zhang[1], MSc; Morgan Broggi[2], MD, PhD; Julia Velz[1], MD; Flavio Vasella[1], MD; Dominik Seggewiss[1], MD; Silvia Schiavolin[3], PsyD; Oliver Bozinov[1], MD; Niklaus Krayenbühl[1], MD; Johannes Sarnthein[1], PhD; Paolo Ferroli[2], MD; Luca Regli[1], MD; Martin N. Stienen[1], MD, FEBNS

[1]Department of Neurosurgery, University Hospital Zurich and Clinical Neuroscience Center, University of Zurich, Frauenklinikstrasse 10, 8091 Zurich, Switzerland

[2]Neurosurgical Unit 2, Department of Neurosurgery, Fondazione IRCCS Istituto Neurologico Carlo Besta, Milan, Italy

[3]Public Health and Disability Unit, Department of Neurology, Fondazione IRCCS Istituto Neurologico Carlo Besta, Milan, Italy

Correspondence to:
Martin N. Stienen, MD / FEBNS
Department of Neurosurgery
University Hospital Zurich & Clinical Neuroscience Center
University of Zurich
Frauenklinikstrasse 10
8091 Zurich, Switzerland
Tel: +41 – (0)44 – 255 – 1111
Email: mnstienen@gmail.com
