# Supplementary material for: Repeated craniotomies for intracranial tumors: is the risk increased? Pooled analysis of two prospective, institutional registries of complications and outcomes
Source: J Neurooncol. 2018 Nov 24;142(1):49–57. doi: 10.1007/s11060-018-03058-y (PMC6399174; doi:10.1007/s11060-018-03058-y)
Supplement: Supplementary file 6 — Online Resource 5 (PDF 73 KB) [file 11060_2018_3058_MOESM6_ESM.pdf]

Online Resource 5: Logistic regression analysis estimating the relationship between repeated microsurgical resection of an intracranial tumor and mortality three months after surgery. The multivariable analysis is adjusted for baseline differences in age, sex, histological diagnosis, size and posterior fossa location of the tumor.

M3 mortality	Univariable analysis			Multivariable analysis		
	OR	95% CI	p-value	aOR	95% CI	p-value
Repeated surgery	0.98	0.58 –1.65	0.936	1.32	0.77 – 2.29	0.307
Age (per year)	-	-	-	1.05	1.03 – 1.06	<0.001
Male sex	-	-	-	1.14	0.74 – 1.74	0.547
Tumor size (per increase in category)	-	-	-	1.62	1.10 – 2.38	0.014
Histology	-	-	-	1.11	1.04 – 1.18	0.002
Posterior fossa location	-	-	-	1.47	0.88 – 2.46	0.138

This online supplementary document is part of the article "Repeated craniotomies for intracranial tumors: is the risk increased? Pooled analysis of two prospective, institutional registries of complications and outcomes" by Costanza Maria Zattra[1,2], MD; David Y. Zhang[1], MSc; Morgan Broggi[2], MD, PhD; Julia Velz[1], MD; Flavio Vasella[1], MD; Dominik Seggewiss[1], MD; Silvia Schiavolin[3], PsyD; Oliver Bozinov[1], MD; Niklaus Krayenbühl[1], MD; Johannes Sarnthein[1], PhD; Paolo Ferroli[2], MD; Luca Regli[1], MD; Martin N. Stienen[1], MD, FEBNS

[1]Department of Neurosurgery, University Hospital Zurich and Clinical Neuroscience Center, University of Zurich, Frauenklinikstrasse 10, 8091 Zurich, Switzerland
[2]Neurosurgical Unit 2, Department of Neurosurgery, Fondazione IRCCS Istituto Neurologico Carlo Besta, Milan, Italy
[3]Public Health and Disability Unit, Department of Neurology, Fondazione IRCCS Istituto Neurologico Carlo Besta, Milan, Italy

Correspondence to:
Martin N. Stienen, MD / FEBNS
Department of Neurosurgery
University Hospital Zurich & Clinical Neuroscience Center
University of Zurich
Frauenklinikstrasse 10
8091 Zurich, Switzerland
Tel: +41 – (0)44 – 255 – 1111
Email: mnstienen@gmail.com
